# Knowledge of HIV and Willingness to Conduct Oral Rapid HIV Testing among Dentists in Xi’an China

**DOI:** 10.1371/journal.pone.0119274

**Published:** 2015-03-05

**Authors:** Lirong Wang, Anthony J. Santella, Ruizhe Huang, Lingling Kou, Lijuan You, Xiaona Zhang, Shu Wang, Jingyao Wang, Longfei Gao, Juan Yin, Guihua Zhuang

**Affiliations:** 1 Department of Epidemiology and Biostatistics, School of Public Health, Xi'an Jiaotong University Health Science Center, Xi'an, Shaanxi, China; 2 Western Sydney Sexual Health Centre, Sydney Medical School, University of Sydney, Parramatta, New South Wales, Australia; 3 Stomatology Hospital, Xi'an Jiaotong University Health Science Center, Xi'an, Shaanxi, China; Emory University Rollins School of Public Health, UNITED STATES

## Abstract

**Introduction:**

China is considered a country of low HIV prevalence (780,000 people living with HIV), however, HIV infections among high-risk populations continue to grow at alarming rates. Voluntary Counseling and Testing services were first implemented in 2003, and oral rapid HIV testing (ORHT) began in 2012. Dentists, as oral health experts, would be well placed to conduct ORHT. We assessed willingness of dentists to undertake ORHT in their clinical practice.

**Methods:**

A cross-sectional, paper-based survey of dentists from the Xi’an region of China was conducted from April to June 2013. Dentists were recruited from Shaanxi Stomatological Association using a stratified sampling methodology. A 40-item survey was used to measure knowledge of HIV, attitudes toward people living with HIV and willingness to conduct ORHT.

**Results:**

477 dentists completed the survey with a mean HIV knowledge test score of 13.2/18 (SD 1.9). If made available in the dental setting, 276 (57.9%) preferred to use blood to diagnose HIV, only 190 (39.8%) preferred saliva or both. Four hundred and thirty-five (91.2%) thought that ORHT was needed in dental clinics. Female dentists felt more accepting of ORHT than males (93.8% vs. 87.8%; χ^2^=5.145; p<0.05). 42.6% of the participants who responded thought that lack of education on ORHT for dentists was the most urgent problem to solve for ORHT, 144 (31.3%) thought that lack of support for ORHT from patients was the most urgent problem. There was statistically significant difference among dental hospital, dentistry and department of dentistry (χ^2^=24.176; p<0.05).

**Conclusions:**

The majority of Chinese dentists thought that ORHT was needed in the dental setting. Providing opportunities for dentists and dental students to learn about HIV testing guidelines and practices is needed as well as feasibility and implementation science research.

## Introduction

Human immunodeficiency virus (HIV)and acquired immunodeficiency syndrome (AIDS) are serious, chronic diseases which have a significant public health burden. In China, 780,000 (range 620,000–940,000) adults and children were estimated to be living with HIV at the end of 2011, with 48,000 (range 41,000–54,000) new infections taking place in 2011, 154,000 (range 146,000–162,000) of the 780,000 were estimated to be living with AIDS [1].

HIV testing is an important public health and health services strategy for controlling the epidemic. People living with HIV (PLWH) are most infectious soon after they become infected and early diagnosis results in both improved clinical and public health outcomes [2]. PLWH who are diagnosed can receive effective treatment to reduce viral loads and morbidity [3, 4], and thus the reduced viral loads may decrease the probability of transmission [5]. Studies indicated that individuals who were aware of their HIV-positive status may be more inclined to modify risky behaviors [6–8]. The HIV/AIDS epidemic can be lessened by increasing the number of PLWH who are aware of their HIV status [6, 9].

Unfortunately, HIV infections are often undiagnosed or diagnosed late [10]. By the end of 2011,cumulative 445,000 PLWH were reported in China, based on the estimates of 780,000 [1], almost half of the PLWH have not yet been tested and diagnosed. One reason for the low HIV testing rate is lack of testing venues. Most of the HIV testing in China is provided by Voluntary Counseling and Testing (VCT) clinics based in Centers for Disease Control (CDCs) and general hospitals [11–13]. To expand testing venues, the Chinese government has explored the feasibility of offering HIV testing in community health centers [14]. To date, this has only been pilot tested.

The dental setting has been found as a logical, clinically relevant venue for HIV testing in the United States (U.S.) [15–18]. Most lesions of HIV infection present orally during the first stages of the disease. And during dental treatment, blood exposure incidents occur frequently, both patients and dentists are at risk of blood-borne infections such as HIV [19, 20]. Dentists in the U.S. have been found to be support of annual HIV testing for high-risk populations. Additionally, being familiar with the Centers for Disease Control and Prevention’s HIV testing guidelines were positively associated with willingness to conduct testing [21]. Dentist’s knowledge and attitudes toward HIV/AIDS reflect this behavior [22]. A qualitative study [23] reported high levels of acceptability in oral rapid HIV testing (ORHT) screening by dental faculty, while another research [17] showed that the dentists were not so actively on ORHT.

Given the lack of prior research on the feasibility of providing ORHT in dental setting in China, we conducted a pilot survey in Xi’an. Xi'an is an inland city in China, which is a traffic hub connecting Yunnan, Sichuan, Xinjiang and other areas with high drug use and known HIV cases. The first case of HIV infection in Xi'an was reported in 1992, and as of October 2012, a cumulative 1,574 HIV/AIDS cases have been reported [24]. Although Xi’an is still an area of low HIV/AIDS incidence and prevalence, HIV infection has been rapidly spreading to the general population [25]. The aim of this study was to explore knowledge of HIV, attitudes toward PLWH and willingness to conduct ORHT among dentists practicing in Xi'an, China.

## Methods

### Study design and participants

A cross-sectional study was conducted to collect data among dentists in Xi’an from April to June 2013. There are three types of dental practice in China. These include: dental hospital, department of dentistry in general hospital and dentistry. Stratified cluster sampling (by type of practice) was used to select participants. Firstly, practices from a list of all dental practices in Xi'an provided by Shaanxi Stomatological Association were divided into three groups according to their types. Next, practices were selected randomly in every type, and then all dentists worked in these selected practices were intended to be surveyed. The dentists were administered surveys in their office between seeing patients. Investigators were trained graduate students. An investigation team including two investigators was responsible for a district. When the dentists finished the questionnaire, the investigators checked whether all the questions were answered, if not, they would remind the dentists to finish all the questions.

A waiver of documentation was approved by the ethics committee, so verbal consent was obtained from each participant. A Research Assistant explained the study to eligible dentists and answered any questions they had prior to asking for their permission to administer the survey. Verbal consent was considered appropriate over written consent because this anonymous study was very low risk and among educated healthcare professionals. All study procedures and consent processes have been approved by the ethics committee of Xi’an Jiaotong University.

### Data collection

The survey instrument was modified from a validated questionnaire from researchers in Seoul, Korea [26]. An expert consensus panel of dental and sexual health scholars reviewed the modifications to conform to the Chinese context of HIV and dentistry. The survey instrument consisted of 40 questions, assessing demographics (4 items), dental practice (7 items), knowledge on HIV/AIDS (16 items), attitude about HIV/AIDS (5 items), attitude toward ORHT (7 items) and perceived barriers to offering ORHT (1 item).

### Analysis

Statistical Package for the Social Sciences (SPSS), version 13.0 was used for statistical analyses. Questions about knowledge on HIV/AIDS contain 16 items and each correct item is awarded one mark; the maximum score is16. Individuals who answered 80% or more of the questions correctly were placed into a category of “high score”, while those who answered less than 80% of the test questions correctly were placed into the “low score” group. Differences between groups were tested using a nonparametric two-tailed Mann-Whitney U test for continuous variables and a chi-squared statistic for categorical variables. Unconditional logistic regression was used to analyze factors influencing knowledge and attitude. Statistical significance was assessed using a two-sided test at the α = 0.05 level for all studies.

## Results

### Participant characteristics

Of the 477 participants, the mean age was 35.4 years (SD 9.6), 205 (43.0%) were males, the mean years in clinical practice was 12.0 (SD 9.8). Seventy-nine percent(n = 377) reported that they had direct contact with infectious diseases by patients’ saliva or blood during the dental procedures, 31.0% of them (n = 117) contacted almost every day. Additionally, 376 (78.8%) had a sharps injury in the last year, 19.1% of them (n = 72) were injured more than 5 times. 412 (86.4%) reported that they possibility treated patients with HIV/AIDS in the last year, but only 20 (4.2%) were sure about that. Other participant characteristics are presented in Table 1.

**Table 1:** Participant Characteristics and HIV Knowledge Score.

	Total(N = 477)	HIV Knowledge Score		χ^2^/z	*P*
		Low Score^a^ (n_1_ = 148)	High Score^b^ (n_2_ = 329)		
Age in years, M (SD)	35.4(9.6)	37.7(11.3)	34.4(8.6)	-2.610	0.009
Gender, n (%)					
Male	205(43.0)	65(31.7)	140(68.3)	0.078	0.780
Female	272(57.0)	83(30.5)	189(69.5)		
Years practiced dentistry, M (SD)	12.0(9.8)	14.6(11.2)	10.7(8.9)	-3.271	0.001
Education, n (%)					
Postgraduate	168(35.2)	38(22.6)	130(77.4)	9.623	0.008
Undergraduate	155(32.5)	51(32.9)	104(67.1)		
College	154(32.3)	59(38.3)	95(61.7)		
Professional title, n (%)					
Professor	89(18.7)	40(44.9)	49(55.1)	12.828	0.002
Dentist-in- Charge	184(38.6)	59(32.1)	125(67.9)		
Dentist	204(42.8)	49(24.0)	155(76.0)		
Type of practice, n (%)					
Dental Hospital	163(34.2)	39(23.9)	124(76.1)	8.349	0.015
Department of Dentistry	157(32.9)	48(30.6)	109(69.4)		
Dentistry	157(32.9)	61(38.9)	96(61.1)		
Contact with blood, n (%)					
Yes	377(79.0)	102(27.1)	275(72.9)	13.254	0.000
No	100(21.0)	46(46.0)	54(54.0)		
Sharp injury in the last year, n (%)					
Yes	376(78.8)	118(31.4)	258(68.6)	0.105	0.746
No	101(21.2)	30(29.7)	71(70.3)		
Possibility to treat HIV/AIDS, n (%)					
Yes	412(86.4)	121(29.4)	291(70.6)	3.885	0.049
No	65(13.6)	27(41.5)	38(58.5)		

^a^answered less than 80% of the test questions correctly;

^b^answered 80% or more of the questions correctly

### HIV knowledge

Given a test of 16 questions on HIV knowledge, the correct answer rates of 12 questions were over 80%, while the other four questions were whether vaginal fluid, being bitten by a mosquito, saliva or mother’s breast milk could transmit HIV, the correct answer rates were 74.2%, 70.9%, 61.4% and 48.2%, respectively. The mean test score of 477 participants was 13.2 (SD 1.9), 148 (31.0%) were in the low score group, 329 (69.0%) were in the high score group.

Knowledge score by different demographic characteristics are shown in Table 1. Younger age, shorter duration of clinical practice, having earned a postgraduate degree, working in a dental hospital, and having direct contact with infectious diseases by patients’ saliva or blood had a higher proportion of high knowledge score. In multiple logistic regression, three independent factors predicted high HIV knowledge score (Table 2). These were: higher education, lower professional title and having direct contact with infectious diseases by patients’ saliva or blood.

**Table 2:** Predictors of HIV Knowledge Score.

	B	SE	Wald	df	*P*	OR	95% CI	
							Lower	Upper
Education			14.265	2	0.001			
Undergraduate	0.611	0.267	5.240	1	0.022	1.842	1.092	3.108
Postgraduate	1.020	0.271	14.126	1	0.000	2.772	1.629	4.717
Professional Title			20.824	2	0.000			
Dentist-in-charge	0.785	0.282	7.741	1	0.005	2.193	1.261	3.815
Dentist	1.381	0.303	20.730	1	0.000	3.981	2.196	7.215
Contact with Blood	0.824	0.241	11.680	1	0.001	2.281	1.421	3.659

### Behavior and attitudes to HIV

Eighty-three percent of participants had never been tested for HIV. When treated patients with HIV/AIDS, 90 (18.9%) chose relieving chief complaint and referring to specialists, 78 (16.4%) chose to treat cautiously in isolated rooms after patient’s agreement, 29 (6.1%) chose the same as non-infected patients. About the likelihood of cross contamination after treatment of HIV/AIDS, 235 (49.3%) thought there was still large risk despite routine disinfection and sterilization. There were no statistically significant differences between those with low and high test scores for the aforementioned statements.

Two hundred and eight six respondents (60.0%) felt comfortable when advising a preliminary positive HIV test result. The high score group felt more comfortable when advising a preliminary positive HIV test result (63.2% vs. 52.7%; χ^2^ = 4.705; *p*<0.05). Multiple logistic regression analyses found that high score participants were more likely than low score participants to feel comfortable when advising a preliminary positive HIV test result (OR = 1.611, 95% CI: 1.706–2.410).

### Attitudes to ORHT

98.7% of participants knew blood could be used to diagnose HIV infection, but only 118 (24.7%) knew saliva could be used to screen for HIV. If made available in the dental setting, 276 (57.9%) preferred to use blood for diagnose HIV, only 190(39.8%) preferred saliva or both.

Four hundred and thirty-five (91.2%) thought that ORHT was needed in dental clinics. There was no statistically significant difference of the attitude to the necessary of ORHT among age, years of practice, education, professional title, type of practice, contact with blood, sharp injury in the last year, and possibility of treating HIV patients. There were no statistically significant difference between those with high and low test scores for the necessary of ORHT (91.8% vs. 89.9%; χ^2^ = 0.473; *p*>0.05). The female participants felt more necessary for ORHT than male (93.8% vs. 87.8%; χ^2^ = 5.145; *p*<0.05) (Table 3). Multiple logistic regression also showed that female participants were more likely to think ORHT is necessary than male participants (OR = 0.480, 95% CI: 0.252–0.915).

**Table 3:** Participant Characteristics and Attitudes toward Rapid HIV Testing.

	Total(N = 477)	Necessity of rapid HIV testing		χ^2^/z	*P*
		Yes (n_1_ = 435)	No (n_2_ = 42)		
Age in years, M (SD)	35.4(9.6)	35.3(9.7)	37.0(9.6)	-1.215	0.224
Gender, n (%)					
Male	205(43.0)	180(87.8)	25(12.2)	5.145	0.023
Female	272(57.0)	255(93.8)	17(6.2)		
Years practiced dentistry, M (SD)	12.0(9.8)	11.7(9.8)	14.3(9.8)	-1.605	0.108
Education, n (%)					
Postgraduate	168(35.2)	153(91.1)	15(8.9)	0.053	0.974
Undergraduate	155(32.5)	142(91.6)	13(8.4)		
College	154(32.3)	140(90.9)	14(9.1)		
Professional title, n (%)					
Professor	89(18.7)	81(91.0)	8(9.0)	0.007	0.997
Dentist-in-Charge	184(38.6)	168(91.3)	16(8.7)		
Dentist	204(42.8)	186(91.2)	18(8.8)		
Type of practice, n (%)					
Dental Hospital	163(34.2)	149(91.4)	14(8.6)	0.014	0.993
Department of Dentistry	157(32.9)	143(91.1)	14(8.9)		
Dentistry	157(32.9)	143(91.1)	14(8.9)		
Contact with blood					
Yes	377(79.0)	347(92.0)	30(8.0)	1.608	0.205
No	100(21.0)	88(88.0)	12(12.0)		
Sharp injury in the last year					
Yes	376(78.8)	342(91.0)	34(9.0)	0.125	0.724
No	101(21.2)	93(92.1)	8(7.9)		
Possibility treat HIV/AIDS					
No	65(13.6)	57(87.7)	8(12.3)		
Yes	412(86.4)	378(91.7)	34(8.3)	1.150	0.284
Knowledge score					
Low score	148(31.0)	133(89.9)	15(10.1)	0.473	0.492
High score	329(69.0)	302(91.8)	27(8.2)		

As seen in Table 4, 42.6% of the participants thought that lack of education on ORHT for dentists was the most urgent problem to solve for offering ORHT, 144 (31.3%) thought that lack of support for ORHT from patients was the most urgent problem. There was significant difference among dental hospital, department of dentistry in general hospital and dentistry (χ^2^ = 24.176; *p*<0.05). Lack of support for ORHT from patients was considered as the most urgent problem in dental hospital, while lack of education on ORHT for dentists was considered as the most urgent problem in dentistry and department of dentistry in general hospital.

**Table 4:** Perceived Barriers to Rapid HIV Testing by Dental Setting, n(%).

	Dental Hospital	Dentistry	Department of Dentistry	Total
Lack of education on rapid HIV testing for dentists	50(31.8)	76(49.0)	70(47.3)	196(42.6)
Lack of support for rapid HIV testing from patients	65(41.4)	39(25.2)	40(27.0)	144(31.3)
Lack of support from health insurance	21(13.4)	21(13.5)	9(6.1)	51(11.1)
Lack of knowledge where to refer HIV infected patients	9(5.7)	9(5.8)	13(8.8)	31(6.7)
Not having enough time to conduct and interpret test	8(5.1)	5(3.2)	12(8.1)	25(5.4)
Not being comfortable giving a positive test result	4(2.5)	5(3.2)	4(2.7)	13(2.8)
Total	157(100.0)	155(100.0)	148(100.0)	460(100.0)

## Discussion

This paper reports the findings of the first Chinese survey of dentists that measured their knowledge, attitudes and willingness to conduct ORHT in the dental setting. Our findings suggest Chinese dentists have a high knowledge of HIV/AIDS. Those dentists who are older, having longer duration of clinical practice, receiving less education, working in private dentistry had lower knowledge scores. Thus, dentist’s knowledge of HIV/AIDS was mainly from school education and the lack of continuing education on HIV/AIDS may possibly lead to the low knowledge score. HIV/AIDS knowledge leads to less discrimination and fear [27, 28], and our findings support this indicating that high score participants were more likely than low score participants to feel comfortable when advising a preliminary positive HIV test result. It is thus necessary to increase the overall knowledge of HIV through either a mentoring program or train-the-trainer model.

It is necessary to further explore the feasibility of ORHT in the dental setting. The majority of participants thought that ORHT was needed in the dental setting, while there are barriers which need to be addressed. Similar acceptability and implementation barriers have been found with dental hygienists in the U.S [16, 29]. These barriers include: educating dental practice staff on HIV/AIDS, how to best continue care for patients after an HIV diagnoses in the dental setting, referring HIV-infected patients to sexual health specialists, dental treatment principles for preventing HIV transmission, HIV test reimbursement and educating staff on pre and post-test counseling [18, 30–32]. A recent U.S. study also found that dentists may overestimate patients’ reluctance to receiving ORHT in the dental setting [21], and thus a patient acceptability study is needed as future research.

Lack of education on ORHT for dentists was identified as the major barrier to ORHT implementation in China. This is not surprising because HIV testing is not taught in dental schools in China and although the dental school curriculum involves infectious diseases, it is taught from a pathophysiology perspective, not diagnostic testing operation. In China, dentists usually do not conduct diagnostic screening tests since these are usually performed by laboratory personnel. ORHT is completed using a relatively simple process. The test could be routinely performed from a patient’s oral fluid in a dentist’s office. It can be predicted dentists are entirely possible competent ORHT after training.

Lack of support for ORHT from patients was identified as second major barriers to ORHT implementation. One reason for patients’ unwillingness to accept HIV testing is pain and fear of blood that comes with traditional blood-based tests [33]. Furthermore, some patients may not be aware of their HIV status because they do not return to the clinic for their test results [34]. ORHT overcomes these hurdles because patients can learn their HIV status in less than 20 minutes, which is within the time frame that a patient is treated by a dental provider. Dietz et al. [35] reported that 73% dental clinic patients would be willing to take a free ORHT during their dental visit.

To date, no HIV or STI guidelines in China have included a role for oral health practitioners, let alone dentists. Dentists, however, do have advanced training in diagnostic skills and patient education, so to expand their role to include ORHT, in select areas of high prevalence, is in the realms of possibility. In conjunction with this, the same government agencies that develop and evaluate prevention and screening guidelines, should consider dentist outreach and social marketing campaigns to not only target dentists but also the general adult population who use their services.

Study limitations include: (a) sample of dentists who participated only practice in one region of China and attitudes may not be generalizable to all Chinese dentists, (b) participants were members of the Shaanxi Stomatological Association which may not capture all practicing dentists, and (c) lack of information on survey non-responders.

Although not a perfect solution to increasing the number of PLWH who are aware of their positive status, the dental setting offers a unique venue for all individuals, regardless of level of perceived risk to test for HIV and other STIs. Dentists, as oral health experts, along with their professional staff (dental hygienists and dental assistants), with proper training and reimbursement could be the additional workforce needed to screen individuals.

This study demonstrates that dentists generally have the knowledge and are willing to conduct ORHT. Additional studies are needed to explore how ORHT can be incorporated into the dental practice setting, methods of reimbursement along with sensitivity and ethical responsibilities of pre- and/or post-test counseling.

## Conclusion

With the introduction of ORHT in China, dental leaders, university training programs, and professional organizations should consider the role of dentists in conducting rapid HIV and other STI testing. Prior to implementation, it will be necessary to educate dentists on HIV testing methods, patient referral and support systems, reimbursement mechanisms, and practice giving positive/reactive results.

## Supporting Information

S1 Dataset (XLS)
